# Endophytic *Bacillus velezensis* XS142 is an efficient antagonist for Verticillium wilt of potato

**DOI:** 10.3389/fmicb.2024.1396044

**Published:** 2024-08-27

**Authors:** Min Li, Jianfeng Yang, Haoyu Li, Yating Wang, Xu Cheng, Guodong Han, Ton Bisseling, Jun Zhao

**Affiliations:** ^1^Laboratory of Molecular Phytopathology, Horticultural and Plant Protection Department, Inner Mongolia Agricultural University, Hohhot, China; ^2^The Modern Agricultural and Animal Husbandry Development Center of Bayannur, Bayannur, China; ^3^Shenzhen Branch, Guangdong Laboratory for Lingnan Modern Agriculture, Genome Analysis Laboratory of the Ministry of Agriculture, Agricultural Genomics Institute at Shenzhen, Chinese Academy of Agricultural Sciences, Shenzhen, China; ^4^Key Lab of Grassland Resources of the Ministry of Education of China, College of Grassland Science, Inner Mongolia Agricultural University, Hohhot, China

**Keywords:** potato Verticillium wilt, *Verticillium dahliae*, *Bacillus velezensis*, biological control, endophytic bacteria, genome sequencing

## Abstract

Potato Verticillium wilt (PVW) caused by *Verticillium dahliae* is a vascular disease, that seriously affects potato (*Solanum tuberosum* L.) yield and quality worldwide. *V. dahliae* occupies the vascular bundle and therefore it cannot efficiently be treated with fungicides. Further, the application of these pesticides causes serious environmental problems. Therefore, it is of great importance to find environmentally friendly biological control methods. In this study, bacterial strains were isolated from agricultural lands on which potato had been cultured for 5 years. Five strains with a broad-spectrum antagonistic activity were selected. Among these five strains, *Bacillus velezensis* XS142 showed the highest antagonistic activity. To study the mechanism of XS142, by which this strain might confer tolerance to *V. dahliae* in potato, the genome of strain XS142 was sequenced. This showed that its genome has a high level of sequence identity with the model strain *B. velezensis* FZB42 as the OrthoANI (Average Nucleotide Identity by Orthology) value is 98%. The fungal suppressing mechanisms of this model strain are well studied. Based on the genome comparison it can be predicted that XS142 has the potential to suppress the growth of *V. dahliae* by production of bacillomycin D, fengycin, and chitinase. Further, the transcriptomes of potatoes treated with XS142 were analyzed and this showed that XS142 does not induce ISR, but the expression of genes encoding peptides with antifungal activity. Here we showed that XS142 is an endophyte. Further, it is isolated from a field where potato had been cultured for several years. These properties give it a high potential to be used, in the future, as a biocontrol agent of PVW in agriculture.

## Introduction

The soil-borne fungus *Verticillium dahliae* Kleb. (*V. dahliae*) causes Verticillium wilt in a wide range of plant species (Fradin and Thomma, 2006; Dhar et al., 2020; Wu et al., 2022). Potato is one of the major crops, whose yield is markedly affected by this disease (Powelson and Rowe, 1993; Rowe and Powelson, 2002). Controlling *V. dahliae* with fungicides is challenging, because once the fungus enters the vascular bundle, the fungicide cannot effectively reach it (Griffin, 2014). It would be attractive to use biological agents that could confer to potato resistance to *V. dahliae*. We hypothesized that bacteria isolated from the potato fields might have the best ability to colonize the root of potato. However, an effective biological agent controlling PVW, isolated from a potato field, has never been described before. Here we selected a collection of bacterial strains that have antagonistic activity to *V. dahliae*. These strains were tested on potato and the strain with the strongest disease suppressing activity has been used to study the underlying mechanism.

*V. dahliae* is a hemibiotroph and it causes a vascular disease (Veronese et al., 2003; Thaler et al., 2004; Tjamos et al., 2005; Lo Presti et al., 2015). The fungus survives in the soil as microsclerotia, which can survive for more than 10 years (Puhalla and Bell, 1981; Short et al., 2015). The germinated hyphae that emerge from microsclerotia penetrate plant roots, pass the cortex and colonize xylem with conidiospores. *V. dahliae* blocks the xylem vessels, interfering with the transportation of water and nutrients. Further, *V. dahliae* produces toxins when it is inside the root (Zhang et al., 2022). Both activities eventually result in foliar stunting, chlorosis, wilting, vascular discoloration, early senescence and ultimately the death of the infected plant (Fradin and Thomma, 2006; Garibaldi et al., 2005; de Sain and Rep, 2015).

Transcriptome analysis is one of the methods to show that plants respond by various mechanisms to infection with *V. dahliae* (Zhang et al., 2020; Guo et al., 2017; Tan et al., 2015). These responses include deposition of lignin, callose, and suberin to delay the infection process, accumulation of antifungal substances, induction of a hypersensitive response (HR) and induced systemic resistance (ISR), etc. (Song et al., 2020; Wu et al., 2022). However, *V. dahliae* can suppress the immune responses of the host plants, making them more susceptible to infection (Gao et al., 2019; Li et al., 2023). Therefore, pesticides are often used, aiming to reduce infection by *V. dahliae*. However, fungicides are not very successful as the fungus inside the plant. Moreover, pesticides have a negative impact on the environment, when they are released into the soil.

It is attractive to use microbes as biocontrol agents because they do not cause environmental problems, but in general the success rate, especially in the field, is limited (Ting, 2014; Deketelaere et al., 2017). It has been argued that the lower success rate is because the biocontrol agents are outcompeted by the endogenous soil microbiome (Ting, 2014). Therefore, it has been proposed that synthetic communities (SynCom) have to be developed that are better able to compete with the endogenous microbiome (Raaijmakers and Mazzola, 2016; Carrión et al., 2019). However, the chance to create such communities would be the best if it starts with strains that have antagonistic activity against the pathogen of interest and are good colonizers of the root of its host. To create such a set of bacteria for potato Verticillium wilt, we made use of the fields on which potato has been grown for several years, chemical fertilizer and fungicides have not been used and Verticillium wilt has occurred.

Here, we describe the isolation of strains from the rhizosphere of potatoes and soil from these fields, their antagonistic activity *in vitro*, their Verticillium wilt suppressing activity in potato and the underlying mechanism were studied by using the bacterium with the strongest suppressing activity.

## Materials and methods

### Soil collection

The potato (Kexin No. 1) roots samples were obtained from the potato fields in Dajing village of Damaoqi, Baotou city (GPS location: N 41° 51′ 40″, E 111° 08′ 10″), Xumayao village, Liangcheng county, Ulanqab city (GPS location: N 40° 48′ 61″, E 113° 34′ 50″) and Hongertu town, Chayouqianqi, Ulanqabu city (GPS location: N 41° 64′ 20″, E 113° 13′ 01″). Rhizosphere and bulk soil samples were each placed in 25 mL phosphate buffer (1 L, NaH_2_PO_4_·H_2_O: 6.33 g; Na_2_HPO_4_·7H_2_O: 16.5 g; Silwet L-77: 200 μL). They were filtered through a 100 μm nylon mesh cell strainer and the filtrate was kept in a 50 mL tube. Glycerol stocks were made for all replicates.

### Bacterial strain isolation

Rhizosphere and bulk soil filtrate were serially diluted (10^−1^, 10^−2^, 10^−3^, 10^−4^, 10^−5^ and 10^−6^) and plated on King’s B medium (KB), supplemented with 100 μg/mL Delvocid to inhibit fungal growth. KB was composed of (g/L): proteose peptone (20 g); MgSO_4_ × 7H_2_O (1.5 g); KH_2_PO_4_ (1.2 g); glycerol (10 mL); agar (15 g). Plates were incubated at 25°C for 3–5 days, and three replicates per dilution were conducted. Bacterial colonies were picked up by sterilized toothpicks. Each colony was purified on a fresh KB plate and subsequently cultured in KB broth. The strains were stored in a 96-well plate at −80°C in 40% glycerol.

### Fungal strain culture

*Verticillium dahlia*e (*V. dahliae*, MN853401), *Fusarium oxysporums* (*F. oxysporum*, MN853482), *Rhizoctonia solani* (*R. solani*) (Liu et al., 2011) and *Phytophthora infestans* (*P. infestans*) (Zhang et al., 2014) were isolated from potato plants in Inner Mongolia. *V. dahliae*, *F. oxysporum* and *R. solani* were cultured in potato dextrose agar (PDA) at 25°C. *P. infestans* was cultured in Rye medium at 18°C.

### High-throughput screening to identify bacterial strains with antagonistic activity

To screen the strains for antagonistic activity, a *V. dahliae* conidial suspension was first made. Briefly, the fresh mycelial PDA plugs (9 mm diameter) of *V. dahliae* were picked from a plate and put into 5 mL 0.9% NaCl. The conidial suspension was filtered through four layers of sterile cheesecloth (grade 50). Spore concentration was determined using a hemocytometer and adjusted to a final concentration of 10^7^ conidia/mL. The conidial suspension of 100 μL was spread on a plate with a diameter of 15 cm. A 96-inoculation needle sterilized with 75% ethanol was used to inoculate the bacterial strains stored in the 96-well plate onto the 15 cm plate. The plates were incubated at 25°C for 3–5 days.

Strains with antagonistic effects were reconfirmed on a 9 cm petri dish inoculated with the conidial suspension. The bacterial strains were grown in 5 mL KB broth overnight at 25°C, bacterial suspension was made with OD_600_ = 1.0, which contains 1 × 10^9^ CFU/mL. The bacterial suspension (OD_600_ = 1.0) of 5 μL was point-inoculated at the periphery of the 1/5th strength PDA plate, and incubated at 25°C for 3–5 days to observe the antagonistic activity (Cheng et al., 2013).

### Screening for strains with broad-spectrum antagonistic activity

A fresh mycelial plug (9-mm diameter) of *F. oxysporum*, *R. solani* or *P. infestans* was placed in the center of a 1/5th PDA plate, 5 μL bacterial suspension (OD_600_ = 1.0) was point-inoculated at the periphery of the 1/5th strength PDA plate. The plates were incubated at 25°C, radial hyphal growth was monitored and the diameter of the inhibition zone was determined. Three plates were used per replication, and the experiment was repeated three times.

### Biocontrol effects of candidate strains against *Verticillium dahliae*

The initial experiments were done with potato tubers. Potato tubers (Holland 15), a Verticillium wilt-susceptible potato variety, were cut into pieces, each with at least one bud eye. A single piece was planted into a plastic pot (10 cm diameter × 10 cm height) filled with a mixture of sterile nursery substrate (peat soil, perlite, vermiculite), sand and soil (1:1:1). To test biocontrol effect, potato seedlings were inoculated at the 4–6 leaves stage (ca. 4–5 weeks after sowing). Five bacterial strains (DS86, XS107, XS142, XS146, XS156) were grown separately in KB broth overnight at 25°C. Bacterial cells were washed with sterile distilled water and re-suspended to make the bacterial suspension (OD_600_ = 1.0). *V. dahliae* was cultured on PDA for 14 days. We inoculated sterilized wheat bran with mycelial plugs. The conidial suspension of *V. dahliae* was made by washing 10-day-old wheat bran culture with sterile water (Zhang et al., 2017). This suspension was filtered through four layers of sterile cheesecloth (grade 50), and was adjusted to 1 × 10^7^ conidia/mL. This conidial suspension was used to inoculate potato plants. Bacterial suspension (OD_600_ = 1.0, 100 mL) of each bacterial strain was poured into a plastic pot with 480 g sterile soil 3 days before inoculation with *V. dahliae*. For the inoculation of SynCom, a mixture of five bacterial strains was made by mixing equal volumes of the five individual suspensions, each with an OD of 1.0. This mixture (100 mL) was added to the pots. The inoculation method of *V. dahliae* was described by Tai et al. (2013). Three 6–8 cm deep holes were made triangularly with a sterile scalpel to wound the root hair, and 100 mL conidial suspension was then poured into three holes. Seedlings were inoculated again 1 week after the first inoculation. Seedlings inoculated only with distilled water were used as mock inoculated seedlings, and ones only inoculated with *V. dahliae* was used as control. The experiment was performed in triplicate, and each treatment within an experiment consisted of 12 replicates. The experiment was conducted in a greenhouse. The seedlings were manually watered every 3 days and no other management measures (e.g., pest or disease control, fertilization) were employed.

The final set of experiment was performed with potato seedlings. Potato tissue culture seedlings (Holland 15) were purchased from Wuchuan County Saifeng Potato Seed Industry Limited Liability Company. The potato virus-free seedling was cut 2.5 cm from the tip and transplanted into a plastic pot (10 cm diameter × 10 cm height) with sterilized vermiculite substrate and Hoagland’s nutrient solution in it. The pots were covered with film for moisture. The film was removed after 5 days of growth, and the potato seedlings with consistent growth were selected after 15 days of transplanting. The bacterial suspension (OD_600_ = 1, 20 mL) of XS142 and XS156 was inoculated around the root of the potato seedlings, separately. Three days after inoculation, 20 mL conidial suspension of *V. dahliae* (1 × 10^7^ conidia/mL) was inoculated around the root again. The potato seedlings inoculated only with *V. dahliae* were used as control. Seedlings inoculated only with distilled water were used as mock. Twelve seedlings were used per replicate and for each treatment 3 replicates were used. The plants were cultured in a climate chamber at 25°C. The water content was 60% of the water holding capacity. The photo period was 16 h/8 h, the light intensity was about 3600 lx.

### Disease assessment and data analysis

Disease severity caused by *V. dahliae* was assessed four weeks after inoculation. Diseased plants were scored with a rating scale designed for potato Verticillium wilt (Rowe, 1985): 0 = no visible symptoms, 1 = leaves with chlorosis especially in older leaves (1–33%), 2 = general chlorosis, and some necrosis and wilting (34–66%), and 3 = severe wilting or death (more than 67%). Disease index (DI) was calculated using the following formula:


$$DI\;=\;\left[\Sigma \;\left(\begin{array}{l} \mathrm{each}\;\mathrm{rating}\;\mathrm{scale}\;\times \;\\ \mathrm{the}\;\mathrm{corresponding}\;\\ \mathrm{number}\;\mathrm{of}\;\mathrm{seedlings} \end{array}\right)/\left(\begin{array}{l} \mathrm{the}\;\mathrm{highest}\;\\ \mathrm{rating}\;\mathrm{scale}\;\times \;\mathrm{the}\;\mathrm{total}\;\\ \mathrm{investigated}\;\\ \mathrm{number}\;\mathrm{of}\;\mathrm{seedlings} \end{array}\right)\right]\;\times \;100.$$

### Fungal transcript level

The transcript level of *V. dahliae* in potato basal stem was studied through real-time quantitative polymerase chain reaction (RT-qPCR). Each of the five individual bacterial strains and the SynCom were inoculated together with *V. dahliae* respectively, and samples were collected 30 days after *V. dahliae* inoculation. The *V. dahliae* transcript level in these 6 treatments were compared with samples from plants inoculated only with *V. dahliae*. RNA was extracted from potato basal stem using the total plant RNA extraction kit (TianGen, Beijing, China). First-strand cDNA was synthesized using reverse transcription kit (TaKaRa, Beijing, China). The specific primer pair of gene *β-Tubulin* (VertBt-F: 5′-AACAACAGTCCGATGGATAATTC-3′; VertBt-R: 5′-GTACCGGGCTCGAGATCG-3′) were used to detect the amount of β-Tubulin mRNA of *V. dahliae*. The potato housekeeping gene *Actin* (Actin-F: 5′-CACCCTGTTCTGCTCACT-3′; Actin-R: 5′-CAGCCTGAATAGCAACATCA-3′) was used as an endogenous control to normalize the samples. Twenty microliters qPCRs were run in three technical replicates on an ABI 7500 Real-Time PCR System (Applied Biosystems), using 2 μL of first-strand cDNAs and 10 μL SYBR Premix Ex Taq (TaKaRa, Beijing, China). RT-qPCR cycles were as follows: one cycle of 30 s at 95°C, followed by 40 cycles at 95°C for 5 s and 60°C for 34 s. Final expression of the target genes was quantified in three repeats using 2^−∆∆Ct^ method.

### Genome sequencing and comparison

Total DNA of strain XS142 was isolated according to the instructions of the bacterial genomic DNA kit (TianGen, Beijing, China). Illumina platform and PacBio RSII technology were combined to sequence the genome of strain XS142. Bacterial genome scans were using short sequence software SOAPdenovo2^1^ and unicycler v0.4.8 software and corrected by Kmer software. The corrected sequences were assembled by Pilon v1.22 software to obtain complete chromosome and plasmid sequences. Glimmer,^2^ GeneMarkS, and Prodigal software were used for prediction of coding sequence of the genome (CDS). The functional classification of the genes of XS142 were performed using COG annotation.^3^ AntiSMASH 7.1.0 software was used to identify the biosynthetic gene clusters (BGCs) related to secondary metabolites. Putative genes encoding for the carbohydrate active enzyme (CAZyme) were identified by CAZyme database.^4^ The genomes of sequenced *Bacillus velezensis* strains were compared with the XS142 genome using the OrthoANI (Average Nucleotide Identity by Orthology).^5^

### Phylogenetic analysis

RNA polymerase beta-subunit (*rpoB*) sequences of *B. velezensis* (CP000560.2, CP006890.1), *B. amyloliquefaciens* (FN597644.1, CP002627.1), *B. subtilis* (NC000964.3), *B. pumilus* (CP027034.1), *B. licheniformis* (NC006270.3) and *B. altitudinis* (CP009108.1, AP025262.1) were obtained from the GenBank genome database. The phylogenetic tree was constructed using the neighbor-joining algorithm and maximum likelihood analyses, with bootstrap values calculated from 1,000 replicate runs using the routines included in MEGA software.

### Colonization of potato roots with XS142-GFP

We followed the methods of Wang et al. (2020) for electrotransformation and transformants screening. The wild type *B. velezensis* XS142 competent cells were prepared as follows. Briefly, single colony of *B. velezensis* XS142 was cultured overnight in 2 mL KB medium at 28°C with 200 rpm/min. Five hundred microliters of the overnight *B. velezensis* XS142 was taken into a 250 mL triangular flask containing 50 mL KB medium at 28°C with 200 rpm/min for 2–3 h. Subsequently, the bacterial cells were centrifuged at 4°C and 5,000 × g for 15 min, followed by addition of a small amount of ultra-pure water (sterilized and pre-cooled). The cells were re-suspended and centrifuged. Then, the bacterial cells were re-suspended with 10% glycerol solution, and 40 μL aliquots of the bacterial cell suspension were placed in each 1.5 mL centrifuge tube. The *B. velezensis* XS142 competent cells were stored at −80°C for future use.

The purified pHT-315 *gfp* plasmid was given by Prof. Zeng of Tarim University. Two microliters of the pHT-315 *gfp* plasmid was added to the competent cells in a 1 mm electrode cup. The electric shock was used with a shock parameter of 1.8 kV for 5–6 ms. One milliliter of SOC liquid medium was added to the electrode cup immediately after the shock, and the cells were resuscitated for 1 h at 28°C with 150 rpm/min. The 200 μL of transformed product was spread on the KB medium with 100 μg/mL erythromycin and cultured overnight in a 28°C incubator. The transformant of XS142-GFP was screened and verified with PCR and double digestion (*ECO*R I+ *Hind* III). XS142-GFP was compared with wild type XS142 in growth rate, genetic stability, fluorescence intensity and antagonistic activity.

Potato virus-free seedlings were immersed in XS142-GFP bacterial suspension (OD_600_ = 1) for 30 min and potato seedlings immersed in sterile water were used as control. Potato roots were analyzed by fluorescence microscopy at 12 hpi, 24 hpi, 3 dpi and 7 dpi, respectively.

### Transcriptome sequencing

To identify genes that are induced by *V. dahliae*, potato seedlings were inoculated with *V. dahliae* and harvested at 3 dpi (Vd_3d). The transcriptomes were compared with water inoculated plants (Mock). To study the effect of XS142 on *V. dahliae* induced genes, seedlings were inoculated with XS142 and 3 days later inoculated with *V. dahliae* (XS142 + Vd_3d), and 3 dpi with *V. dahliae* (Vd_3d) plants were harvested (This is 6 dpi with XS142, XS142_6d). Three biological replicates were performed. RNA sequencing was performed at Shanghai Meiji Biomedical Technology Co., Ltd. Total RNA was isolated and cDNA libraries were constructed using the Illumina Truseq^TM^ RNA sample prep Kit. RNA sequencing was performed with the Illumina Novaseq 6000 platform. The reference gene source was *Solanum tuberosum* with a reference genome version of DM_v6.1. Reference genome source: http://solanaceae.plantbiology.msu.edu/index.shtml. In RNA-Seq analysis, gene expression levels were calculated by the number of clean reads (reads counts) that were located in genomic regions. The quantitative expression results were based on TPM. These values were calculated and used to estimate the effects of sequencing depth and gene length on the mapped read counts.

All Genes and transcripts obtained by transcriptome assembly were compared with six databases: Non-redundant (NR), SwissProt, Kyoto Encyclopedia of Genes and Genomes (KEGG), Clusters of Orthologous Groups (COG), EggNOG (Evolutionary Genealogy of Genes: Non-supervised Orthologous Groups) and Pfam.

### Statistical analysis

The experiments with potato were performed with three replicates and they were grown in a randomized block design. The data related to biocontrol effects are disease index and relative amount of *V. dahliae*. They were analyzed by analysis of variance (ANOVA), and the significance of differences was determined by Duncan’s new multiple range test at the 5% (*p* < 0.05) level of significance between treatments in SAS (version 9.4). Error bars represent the SD (standard deviation) of the three replicates.

In RNA-Seq analysis, the DESeq2 program^6^ was used for the differential expression gene analysis. Genes with the expression level >25 TPM, the false discovery rate (FDR) *p*-value <0.05, and a fold change of >5 were used as criteria to select genes induced by *V. dahliae* (comparison of Vd_3d with Mock). To select the genes that were induced by XS142, the genes with an expression level >25 TPM, the FDR *p*-value <0.05, and a fold change >4 were used as criteria (comparison of XS142_6d with Mock).

## Results

### High-throughput screening for strains from potato with antagonistic activity against *Verticillium dahliae*

We aimed to create a collection of bacteria with antagonistic activity against *V. dahliae* and three other potato pathogens, such as *Fusarium oxysporums*, *Rhizoctonia solani* and *Phytophthora infestans*, which caused Potato Root Rot, Potato Black Scurf and Potato Late Blight respectively.

**Figure 1:**
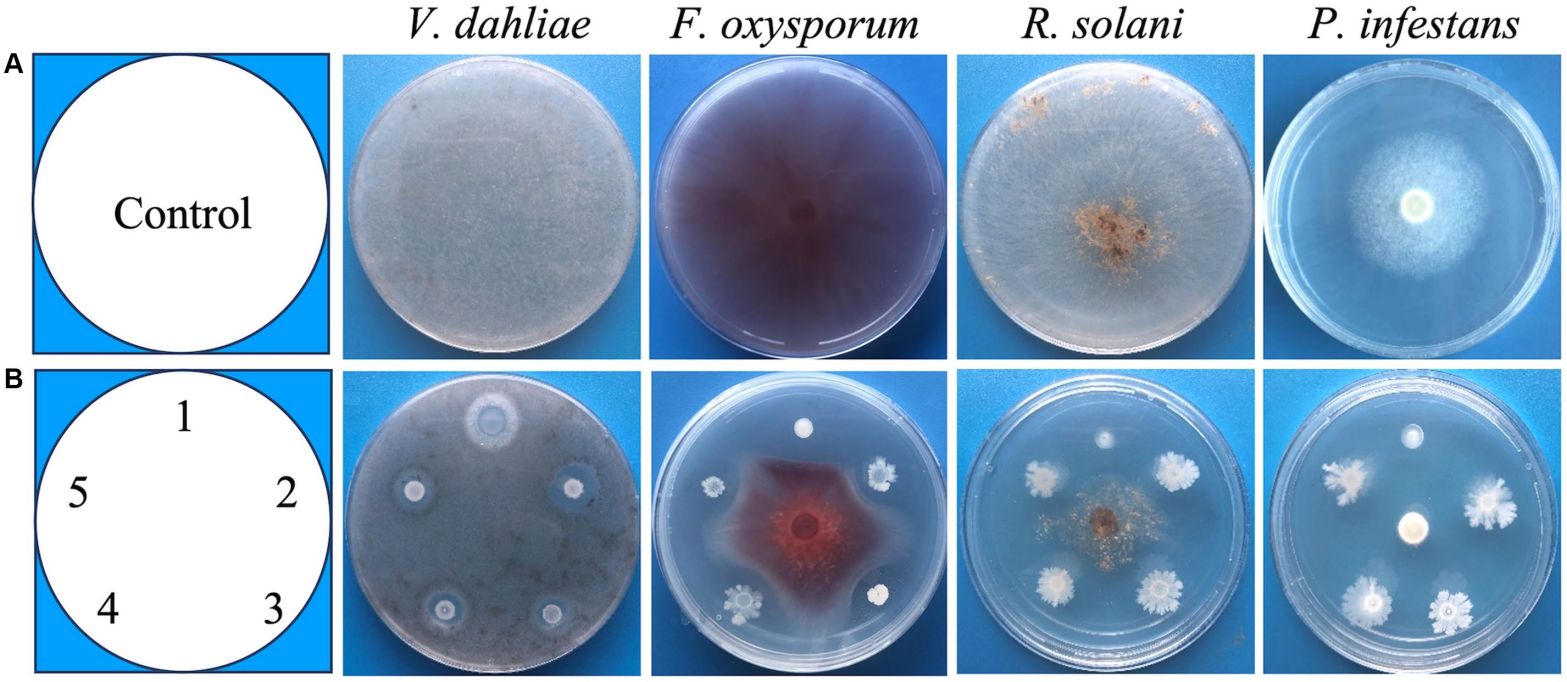
Screening of broad-spectrum antagonistic strains. **(A)** The growth of *V. dahliae* (*Verticillium dahliae*), *F. oxysporum* (*Fusarium oxysporums*), *R. solani* (*Rhizoctonia solani*) and *P. infestans* (*Phytophthora infestans*), in the absence of antagonistic strains (Control). **(B)** Co-culture of fungi and antagonistic strains: 1 = DS86, 2 = XS146, 3 = XS156, 4 = XS107, 5 = XS142.

We isolated bacteria from fields on which potato had been grown for 5 years and chemical fertilizer and fungicides were not applied. A total of 1,456 isolates were isolated from rhizosphere of potato and bulk soil from these fields. These isolates were screened for their antagonistic activity against *V. dahliae* by a high-throughput screening method on plates (see materials and methods). Strains that created a halo around the bacteria were selected. To select the strains with a pathogen suppression capacity, similar plate assays with *F. oxysporums*, *R. solani* and *P. infestans*, respectively, were performed (Figure 1). Finally, 5 strains with antagonistic activity against all four pathogens were selected. These strains were named DS86, XS107, XS142, XS146 and XS156. The antagonistic activity of all 4 pathogens was semi-quantified by measuring the diameter of the inhibition zone (Table 1). The inhibition diameters for *V. dahliae* were between 0.53 cm (XS142) and 0.93 cm (XS146).

**Table 1:** The diameter of the inhibition zone with antagonistic activity.

Strains	*V. dahliae* (cm)	*F. oxysporum* (cm)	*R. solani* (cm)	*P. infestans* (cm)
DS86	0.80 ± 0.05	1.12 ± 0.21	1.38 ± 0.08	2.88 ± 0.38
XS107	0.92 ± 0.03	1.52 ± 0.05	1.35 ± 0.30	4.70 ± 0.66
XS142	0.53 ± 0.06	0.80 ± 0.23	1.63 ± 0.21	3.00 ± 0.20
XS146	0.93 ± 0.06	0.77 ± 0.19	2.17 ± 0.50	2.73 ± 1.60
XS156	0.63 ± 0.10	0.43 ± 0.14	1.17 ± 0.46	3.52 ± 0.38

### Biocontrol effect on *Verticillium dahliae*

**Figure 2:**
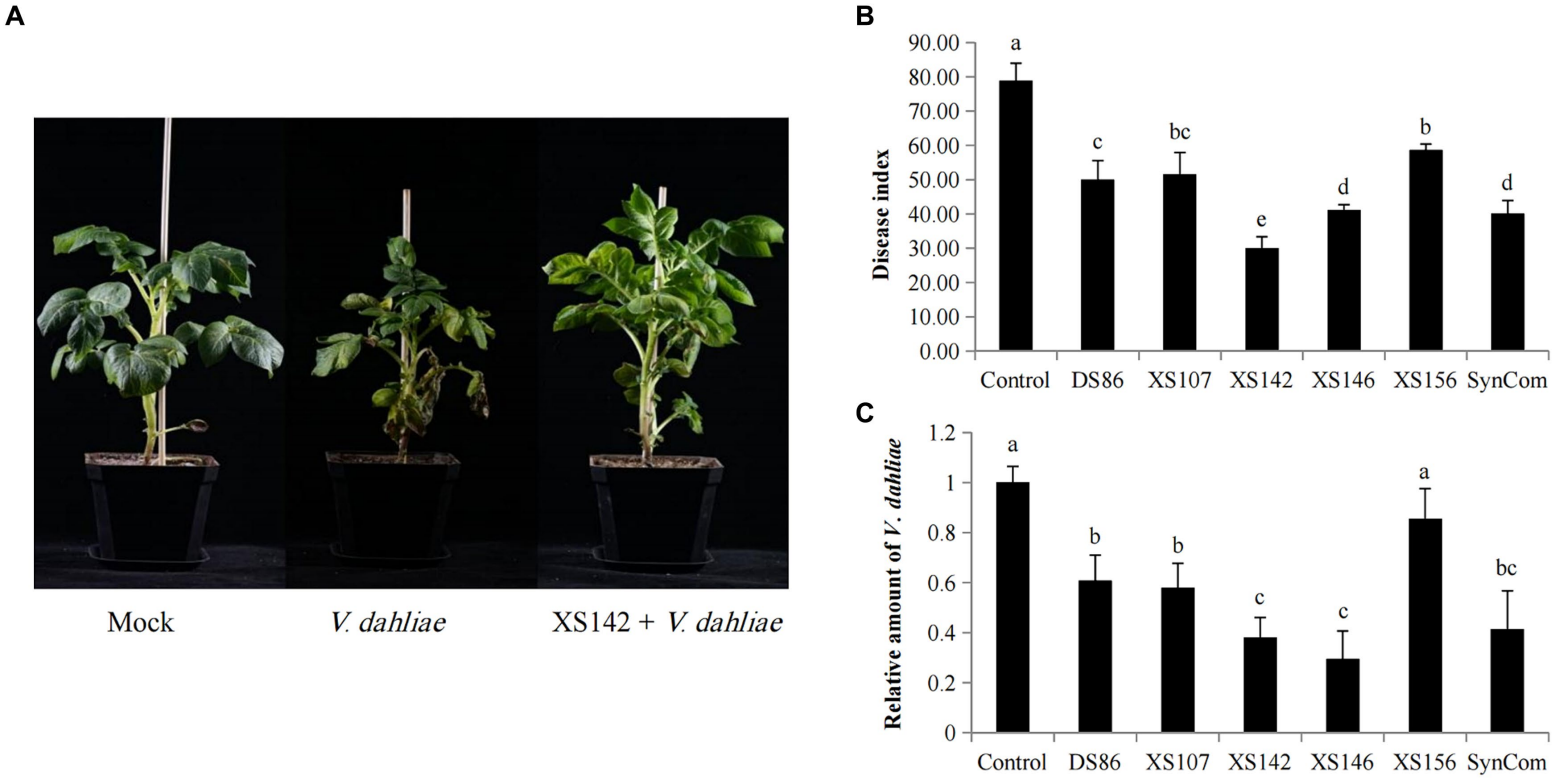
Biocontrol effect of potato strains on potato Verticillium wilt. **(A)** The plant phenotypes of mock, control and the co-inoculated by XS142 and *V. dahliae* 30 dpi. **(B)** Disease index of the infected plants at 30 dpi. Significance of differences was determined by Duncan’s new multiple range test (*p* < 0.05). Error bars represent the SD (standard deviation) of the three biological replicates. **(C)** Real-time quantitative PCR (RT-qPCR) to determine the relative amount of *V. dahliae* in potato stems. The *β-tubulin* gene was used to quantify *V. dahliae*, and the potato *Actin* gene was used to normalize the data. Significance of differences compared to control were determined by Duncan’s new multiple range test (*p* < 0.05). Error bars represent the SD of the three biological replicates.

The antagonistic effect of the individual strains and that of the SynCom on potato were shown in Figure 2. All the inoculated potatoes have symptoms of Verticillium wilt. The initial wilt symptoms are yellowing of the oldest leaves (Figure 2A). After 30 days post-inoculation (dpi), the control showed a severe wilting and necrosis (Figure 2A). However, the plants co-inoculated with the strains and SynCom had less severe symptoms. To quantify the biocontrol effect, the disease index was calculated (see materials and methods) (Figure 2B).

The disease index of the control was 78.89 and those of the plants inoculated with a single strain ranged from 30.0 (XS142) to 58.64 (XS156) and that of plants treated with the SynCom was 40.08 (Figure 2C). So, all 5 strains, as well as the SynCom, showed biocontrol effect on *V. dahliae*. The most effective one, XS142, reduced the disease index significantly better than the SynCom (Figure 2B). In Figure 2A it was shown that at 30 dpi with *V. dahliae*, the size of potato plants was markedly smaller in comparison with the mock inoculated plants, whereas the size of plants co-inoculated with XS142 was similar to that of mock inoculated plants.

To quantify the suppression of fungal growth, we determined the relative amount of *V. dahliae in planta* by quantifying β-Tubulin mRNA level by RT-qPCR (Figure 2C). The fungal transcript level was most reduced by co-inoculation *V. dahliae* with strains XS142 and XS146, separately. This reduction was 62.03 and 70.59%, respectively, in comparison to plants only inoculated with *V. dahliae*. Except for the plants treated with XS156 strain, the other strains also reduced fungal transcript level significantly, albeit less efficient than strains XS142 and XS146.

For further studies we selected strain XS142 for the following reasons: strain XS142 showed a stronger reduction of the disease index than strain XS146, whereas the reduction of the fungal transcript level by the 2 strains was not significantly different. Furthermore, the reduction of the disease index by treatment with the SynCom, was less than that by strain XS142.

### XS142 is a *Bacillus velezensis* strain

To determine the identity of XS142, we determined its genome sequence. XS142 has a size of 4,087,900 bp and a GC content of 46.39% (Table 2). Gene prediction showed that there were 3,855 protein coding sequences (CDSs), and 87 tRNA and 27 rRNA genes. A total of 3,046 genes were classified into 20 functional categories of Clusters of Orthologous Groups (COGs). The COGs annotation showed that the largest category included genes of unknown function (783 genes), followed by genes involved in transport and metabolism of amino acids (288 genes), and genes involved in transcription (239 genes) (Supplementary Figure S1).

**Table 2:** The general genome features of XS142 and FZB42.

Genome feature	XS142	FZB42
Genome size (bp)	4,087,900	3,918,596
G + C content (%)	46.39	46.5
Protein-coding sequences	3,855	3,680
rRNAs	27	29
tRNAs	87	88
Genes assigned to COG categories	3,046	2,993

**Figure 3:**
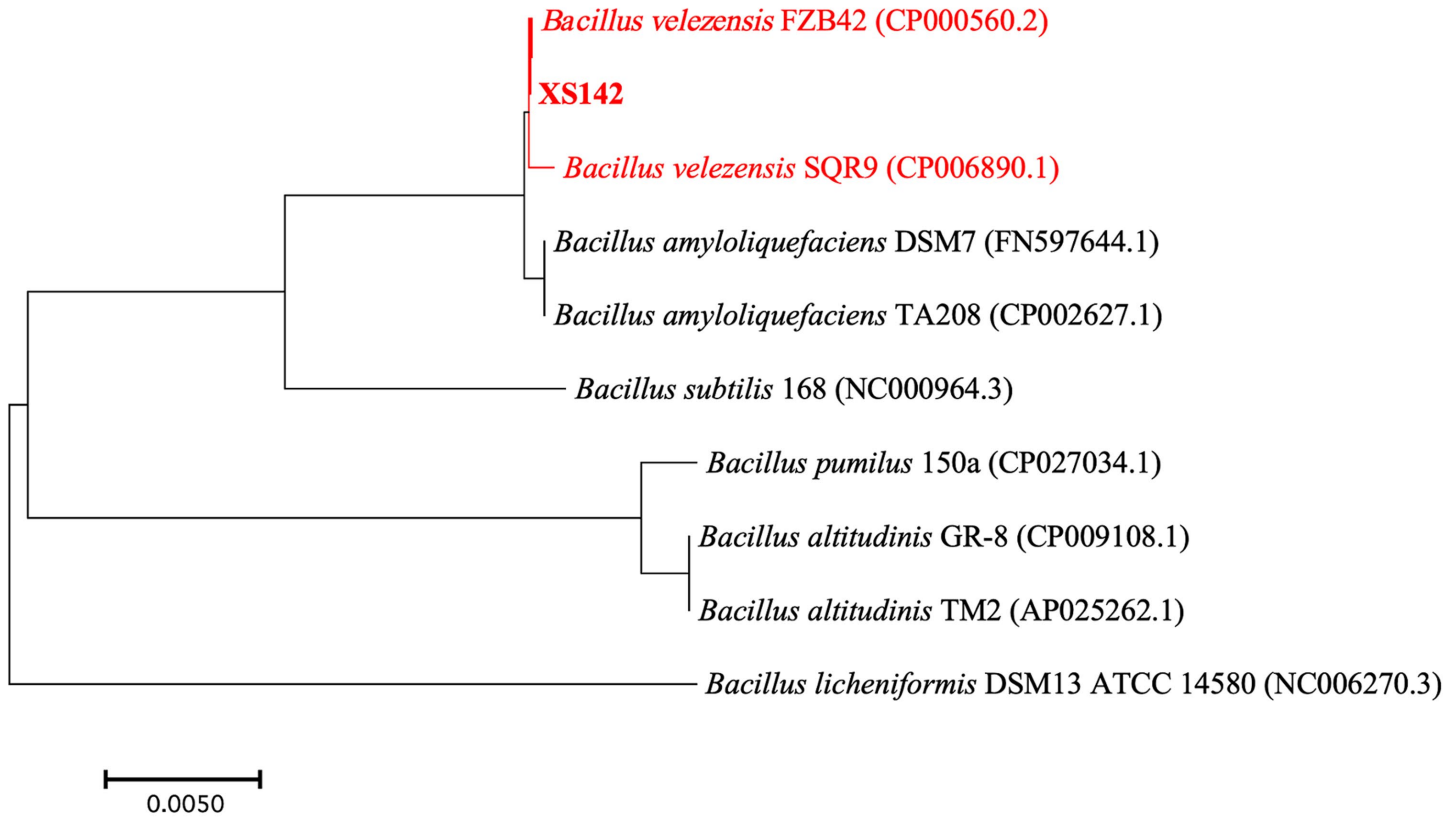
Phylogenetic relationship of *rpoB* genes of *Bacillus* species. Phylogenetic tree constructed using *rpoB* gene sequences of type strains of Bacillus species by the neighbor-joining method (using MEGA software). Bootstrap values >50%, are based on 1,000 repetitions. *B. velezensis* FZB42 (CP000560.2), *B. velezensis* SQR9 (CP006890.1), *B. amyloliquefaciens* DSM7 (FN597644.1), *B. amyloliquefaciens* TA208 (CP002627.1), *B. subtilis* 168 (NC000964.3), *B. licheniformis* DSM13 ATCC (NC006270.3), *B. pumilus* 150a (CP027034.1), *B. altitudinis* GR-8 (CP009108.1), *B. altitudinis* TM2 (AP025262.1) are indicated at branch points.

We analyzed the GenBank databases using the 16S rRNA gene sequence of XS142. This result showed XS142 belongs to the genus *Bacillus*. To identify to which species XS142 is closest related, a phylogenetic tree was made by using RNA polymerase beta-subunit (*rpoB*) sequences (Figure 3). The *rpoB* sequences of *B. velezensis*, *B. amyloliquefaciens*, *B. subtilis*, *B. pumilus*, *B. licheniformis* and *B. altitudinis* were obtained from the GenBank genome database. This tree showed that strain XS142 is closest related to *B. velezensis* species. One of them is *B. velezensis* FZB42, which is a PGPR model strain.

To determine whether XS142 is a *B. velezensis* species we used OrthoANI (Average Nucleotide Identity by Orthology). OrthoANI is an algorithm and software to determine average nucleotide identity (Lee et al., 2016). OrthoANI is used for species demarcation of bacteria and values >95% are used to show that bacterial strains are the same species (Goris et al., 2007; Richter and Rosselló-Móra, 2009; Lee et al., 2016). The OrthoANI values of XS142 was determined by comparing it to strains of *B. velezensis*. The OrthoANI value of FZB42 (CP000560) was 98.31%, followed by SQR9 (CP006890.1) 98.27%. The OrthoANI value of *B. amyloliquefaciens* DSM7 (FN597644.1) and TA208 (CP002627.1) are both 94.14%, so less than 95%. Based on the phylogenetic and the OrthoANI analysis, XS142 was classified as *B. velezensis*. The genome sequence of XS142 was submitted to NCBI GenBank under Accession Number CP148896.

### Colonization of potato roots by XS142

**Figure 4:**
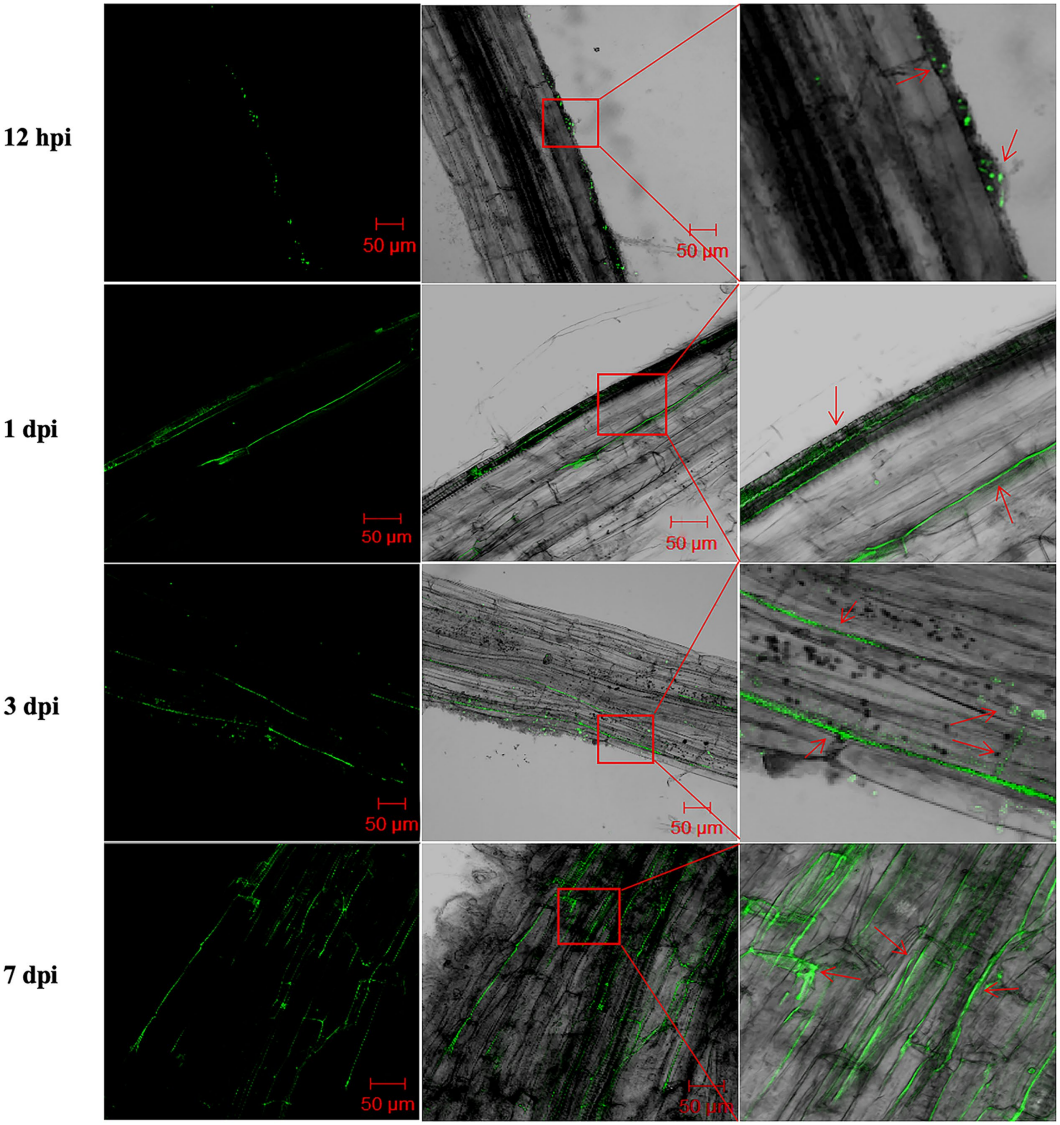
Root colonization by *B. velezensis* XS142-GFP. Roots were analyzed at 12 hpi, 24 hpi, 3 dpi and 7 dpi. Left column represents the fluorescence microscopic observations of the roots at different time points after inoculated with *B. velezensis* XS142-GFP. The middle column shows the merged bright field and fluorescence microscopy pictures. The right column contains the magnifications of the red box areas in middle column. The red arrows in the right column mark the location of *B. velezensis* XS142-GFP.

It has been proposed that endophytic bacteria with antagonistic activity will be more effective against vascular pathogens than rhizosphere bacteria (Eljounaidi et al., 2016). Therefore, we studied the mode of root colonization by XS142. To do this, XS142 was transformed with a GFP construct. XS142 expressing GFP (XS142-GFP) was shown to maintain its antagonistic activity (Supplementary Figure S2). Potato seedlings inoculated with XS142-GFP were analyzed at 12 hpi (hours post-inoculation), 1 dpi, 3 dpi and 7 dpi (Figure 4). At 12 hpi, green fluorescence dots could be observed on the root surface; at 1 dpi, it was distributed along the root epidermal intercellular space; at 3 dpi it occurred in the cortical intercellular space; and at 7 dpi the green fluorescence signal formed a network in the root cortex. So strain XS142 is an endophyte, as it can colonize the interior of potato roots.

### Biocontrol activity of XS142 and XS156 in virus-free potato seedlings

In order to study whether XS142 can induce resistance mechanisms, we first established a virus-free potato seedling system, because the presence of virus might already induce resistance mechanisms. In the above-described experiments, we used seedlings produced by tubers, but it cannot be excluded that some other pathogens, like virus, are present in the tubers. Further, there is an additional disadvantage, because the size of seedlings can be affected by tuber size. Therefore, in the following experiments, we made use of seedlings generated by tissue culture, as these results in seedlings with similar size and they are virus-free (see materials and methods).

**Figure 5:**
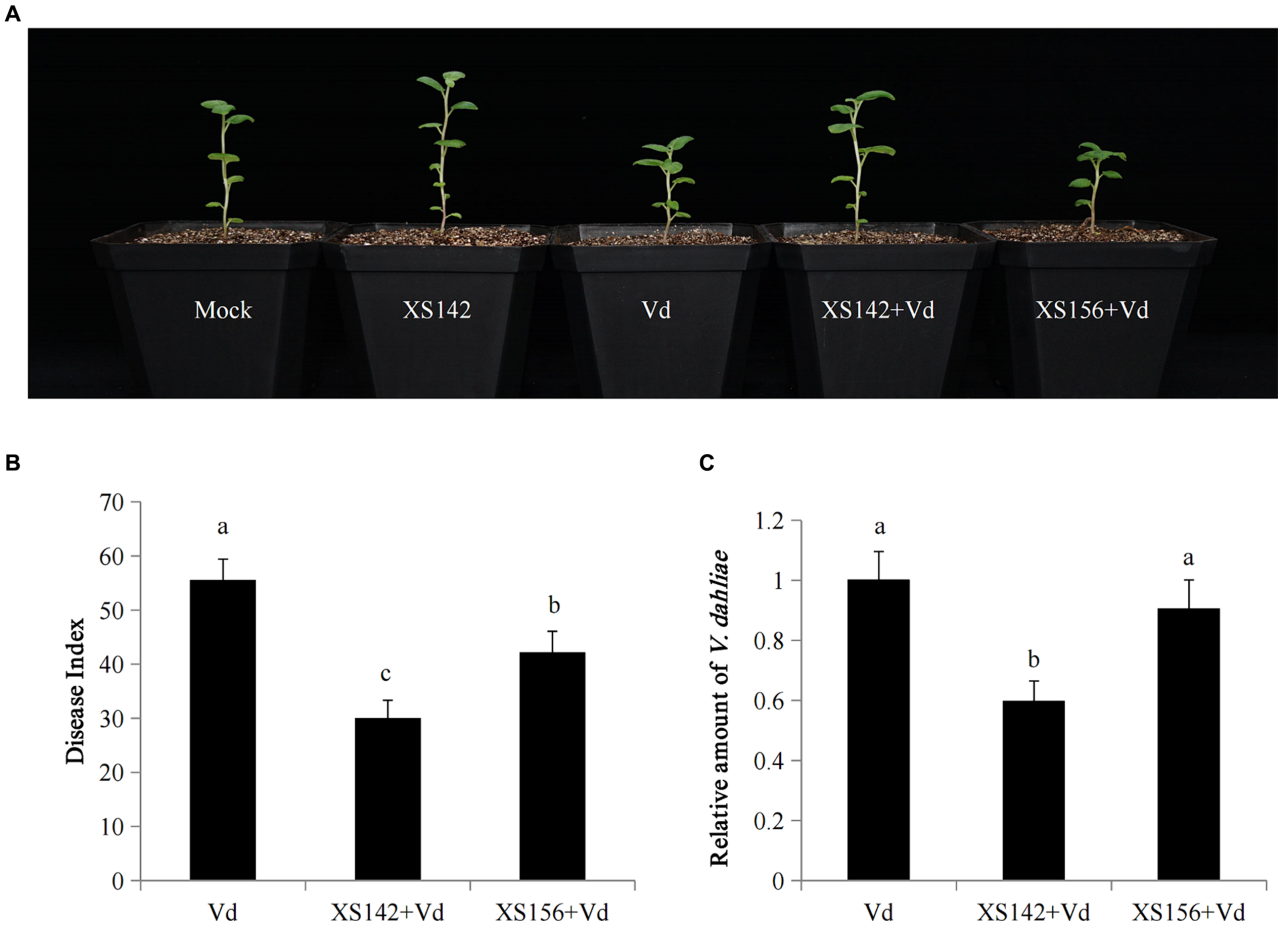
Biocontrol effects of XS142 on potato Verticillium wilt. **(A)** Mock, only inoculated with water, Vd: only inoculated with *V. dahliae*, XS142 + Vd or XS156 + Vd: pretreated with XS142 or XS156 and then inoculated with *V. dahliae*. Photographs were obtained at 10 dpi with *V. dahliae*. **(B)** Disease index. **(C)** Quantitative analysis of the transcript level of *V. dahliae*.

As a first step we studied whether strain XS142 confers resistance to *V. dahliae*, when we used these potato virus-free seedlings (Figure 5A). As a comparison, we also used strain XS156, which had a much lower biocontrol activity in the former experiments. Potato seedlings of similar size were selected (see materials and methods) and were grown on sterilized vermiculite. These seedlings were inoculated with the biocontrol strains or mock inoculated. After 3 days of growth, *V. dahliae* conidial suspension (20 mL × 10^7^ conidia/mL) was added.

We determined the disease index and quantified the relative amount of fungus by RT-qPCR after 30 dpi with *V. dahliae* (Figures 5B,C). The disease index of plants inoculated with strain XS142 or XS156 was 30.0 and 42.22, respectively, whereas in the absence of these strains the disease index was 55.66. So the disease index was reduced with 46 and 24%, which was similar as in the former experiment (Figure 2). In both cases, the difference with the only *V. dahliae* (control) inoculated plants was significant. The quantification of the relative amount of *V. dahliae*, showed that inoculation with XS142 had led to a reduction of 40.15% in comparison to the control, whereas inoculation with XS156 reduced the amount of fungus with only 9.41%. Further, the difference between control and inoculation with XS156 was not significant.

So, the established virus-free potato seedling system can be used to study the mechanism by which XS142 confers resistance to *V. dahliae*.

### The mechanism of antagonistic activity of XS142 on *Verticillium dahliae*

To determine the putative underlying mechanism of XS142 antagonistic activity on *V. dahliae*, we first compared the genome sequence of XS142 with that of *B. velezensis* FZB42 (Table 2). FZB42 is a model strain, of which the mechanisms by which it suppresses plant pathogenicity and stimulates plant growth have been well studied (Krebs et al., 1998; Chen et al., 2007, 2009; Borriss et al., 2019; Rabbee et al., 2019).

**Figure 6:**
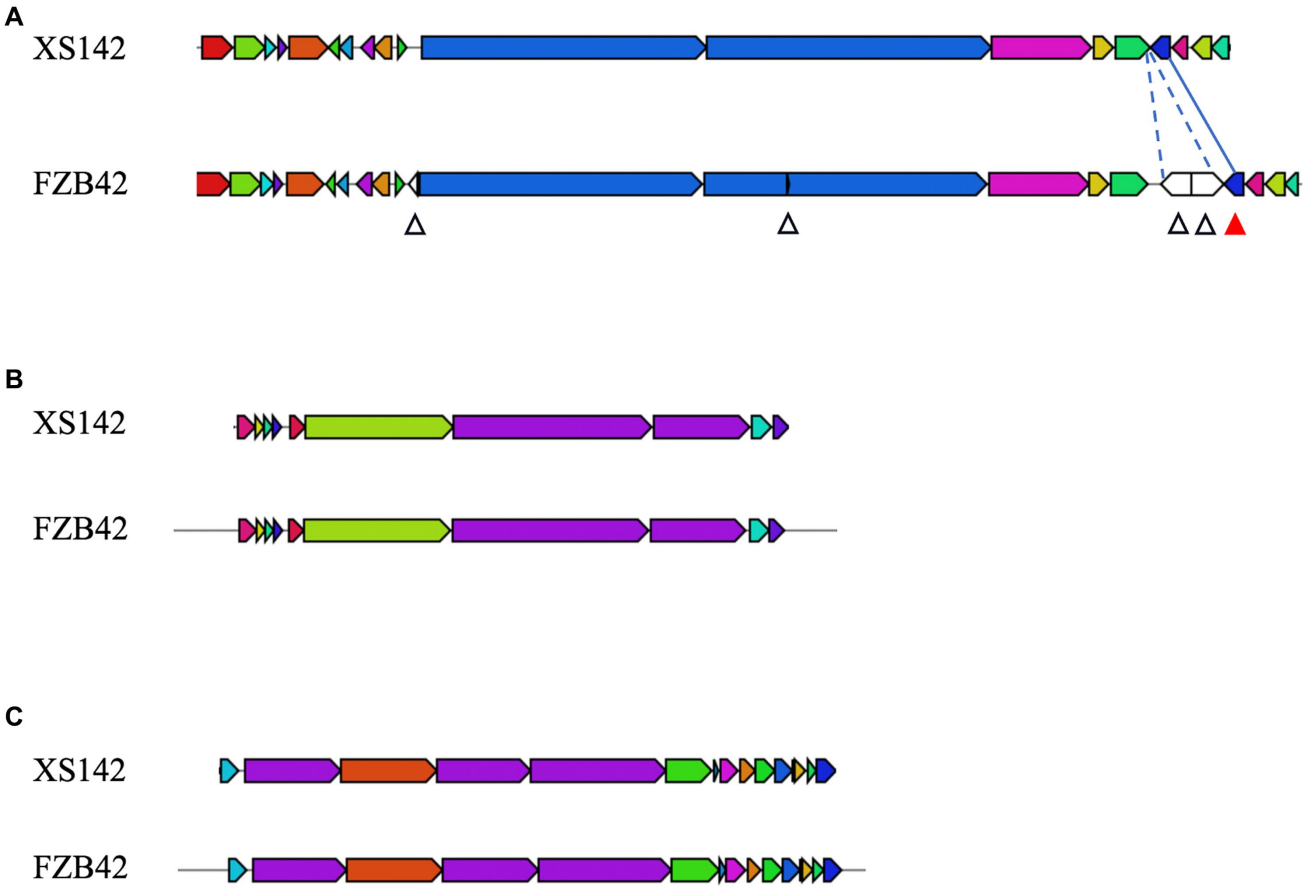
Comparison of biosynthetic gene clusters involved in production of antifungal metabolites. Each block represents a gene, XS142 and FZB42 were compared for the presence of these genes. **(A)** Surfactin BGC. Four genes are lacking in XS142 therefore 82% similarity (with respect to presence of genes) with FZB42. The white arrow heads indicate the genes in FZB42 that are lacking in XS142. The red arrow head indicate the gene (*sfp*) in FZB42 that is present in XS142. **(B)** Fengycin, 100% similarity. **(C)** Bacillomycin D, 100% similarity.

The genome of FZB42 harbors 13 biosynthetic gene clusters (BGCs) that are involved in the biosynthesis of the compounds with antimicrobial activity. This result is summarized in Table 3. Eleven of these clusters are present with a high level of sequence identity in the genome of XS142. Three of them have been demonstrated to have a biocontrol effect on fungi. These are the clusters for the production of surfactin, bacillomycin D and fengycin, respectively. The genes of these 3 clusters of XS142 are compared with those of FZB42 in Figure 6. In case of the bacillomycin D and fengycin clusters, all (100%) of the genes are shared (Figures 6B,C). The surfactin BGC of FZB42 contains 22 genes of which 4 genes are lacking in the genome of XS142 (82% identity) (Figure 6A). However, the core biosynthetic genes, *srfABCD*, all are present in the XS142 genome.

**Table 3:** Genes and BGCs encoding for secondary metabolites in *B. velezensis* FZB42 and XS142.

MIBiG accession	FZB42 BGCs metabolites	Biocontrol effect	XS142 BGCs metabolites	Similarity^a^
BGC0000433	Surfactin	Fungal	+	82%
BGC0001090	Bacillomycin D	Fungal	+	100%
BGC0001095	Fengycin	Fungal	+	100%
BGC0001184	Bacilysin	Bacterial	+	100%
BGC0000569	Plantazolicin	Bacterial	+	91%
BGC0001185	Bacilibactin	Siderophore production	+	100%
BGC0000181	Macrolactin	Bacterial	+	100%
BGC0001089	Bacillaene	Bacterial	+	100%
BGC0000176	Difficidin	Bacterial	+	100%
BGC0000616	Amylocyclicin	Bacterial	+	100%
—	Antibacterial peptide	Bacterial	+	−
—	Orphan	—	—	—
—	Triketide pyrone	−	—	—

a % of genes that are shared.

In FZB42 the coding region of *ComS* is located within that of one of the core genes. This coding region is not present in the gene of XS142. ComS is a regulatory protein involved in the level of surfactin production.

The *sfp* gene has previously been shown to be important for the synthesis of non-ribosomal lipopeptides and polyketides (Chen et al., 2009). This gene is present in the surfactin gene cluster of XS142 with a similar position as in the cluster of the model strain (Figure 6A). Further, this gene shares 98.6% sequence identity with that of FZB42. So, a surfactin most likely still can be made by XS142, but the production of surfactin may be reduced, and its structure might be slightly different. So XS142 harbors at least 3 BGCs encoding for antifungal secondary metabolites.

FZB42 also has the ability to suppress fungal growth by the production of several volatiles (Borriss, 2011; Tahir et al., 2017; Borriss et al., 2019). However, the genome of XS142 only shares the genes encoding the following proteins involved in volatile biosynthesis: acetolactate synthase (AlsS) (99.8% sequence identity at amino acid level), acetolactate decarboxylase (AlsD) (100%) and acetoin reductase (99.7%).

Furthermore, chitinase is a key hydrolytic enzyme that degrades chitin of the fungal cell wall. XS142 has a chitinase gene as was shown by analyzing the CAZymes database. Using the XS142 chitinase sequence, we showed that FZB42 has a similar gene with 99.4% sequence identity at the amino acid level.

In conclusion, XS142 most likely has the potential to suppress the growth of *V. dahliae* by bacillomycin D, fengycin, surfactin and chitinase. In addition, XS142 has BGCs that are not shared with FZB42 or are not 100% identical. Therefore, it cannot be excluded that XS142 produces additional compounds with antifungal activity.

### Transcriptome analysis

**Figure 7:**
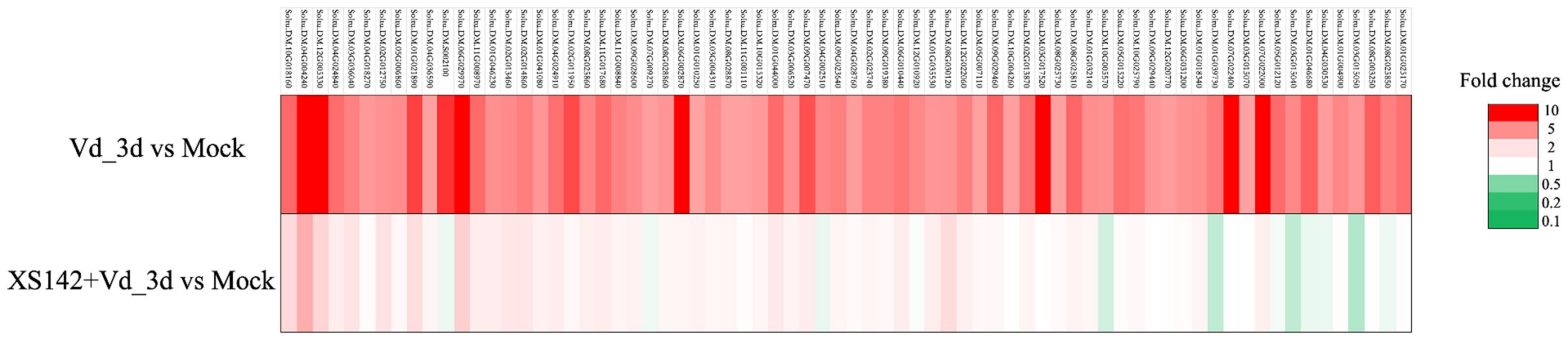
The effect of XS142 on the expression level of *V. dahliae* induced genes. Seventy-two genes were selected which were >5 fold induced (Vd_3d vs. Mock). The expression of all these genes was markedly reduced by pretreatment with XS142 (XS142 + Vd_3d vs. Mock).

It has been shown that several compounds of FZB42 are required for the induction of ISR (Chowdhury et al., 2015; Fan et al., 2018; Borriss et al., 2019). To determine whether ISR is induced by XS142, we first identified *V. dahliae* upregulated potato genes. Samples were collected from *V. dahliae* treated plants at 3 dpi. Seventy-two genes were identified that are >5 fold upregulated (Supplementary Table S1). These genes were used as marker to test whether pretreatment with XS142 resulted in priming for defense. All the 72 genes were markedly less upregulated in plants co-inoculated with XS142 and *V. dahliae* than in plants only treated with *V. dahliae* (Figure 7). The expression level of the genes showed that pretreated by XS142 did not result in an enhanced transcriptional response upon inoculation with *V. dahliae*. Therefore, we conclude that XS142 does not induce ISR and the reduced level of *V. dahliae* in XS142 pretreated plants must have a different cause.

To determine whether XS142 activates defense related genes of potato, in total 7 genes were identified (Table 4), among them, 3 genes that encode for an osmotin. These genes are part of the PR-5 family (thaumatin-like protein) with antifungal properties (Chowdhury et al., 2017). The other four genes encode cellulose synthase-like D3, jasmonate-zim-domain protein, PAR1 protein, and a hypothetical protein. However, their function in defense is not clear. Based on the induction of expression of the osmotin genes, we conclude that XS142 enhances the antifungal activity of potato.

**Table 4:** Potato genes activated by XS142.

Sequencing ID	Gene description	Fc (XS142/mock)
Soltu.DM.03G020780	Cellulose synthase-like D3	4.70
Soltu.DM.12G026270	Jasmonate-zim-domain protein	4.98
Soltu.DM.03G017710	PAR1 protein	4.65
Soltu.DM.04G026480	Hypothetical protein	9.07
Soltu.DM.08G027240	Osmotin	6.20
Soltu.DM.11G016790	Osmotin	5.61
Soltu.DM.08G027280	Osmotin	4.06

## Discussion

We demonstrated that the bacterium XS142 is a *Bacillus velezensis* strain. This strain was isolated from a fungicide-free potato field and has the highest antagonistic activity against *V. dahliae in planta*. *B. velezensis* XS142 markedly reduces the colonization level of *V. dahliae* in potato. This strain has a high potential to directly antagonize *V. dahliae* by synthesizing compounds with antifungal activity. Further, it is shown that XS142 activates potato genes encoding peptides with antifungal activity.

We analyzed the antagonistic activity of 5 strains as well as a SynCom made of these 5 strains. In this study, the antagonistic activity against *V. dahliae* was used, whereas the antagonistic activities against other pathogens will give the collection a higher potential to be used as biocontrol agent and these strains can also support future studies on these potato pathogens.

Two strains XS146 and XS142 had a rather similar high antagonistic activity against *V. dahliae* in potato plants. We selected XS142 for more detailed analysis, as it was the strain which reduced the disease index most efficient. However, in future research we will analyze the antagonistic activity of XS146 in more detail. As individual strain XS142 was also more effective than the SynCom including this strain. It remains to be studied why this is the case. It will be important to study the population dynamics of the 5 strains *in planta*, as it is possible that strains with a lower antagonistic activity are better colonizers of potato roots and outcompete the more efficient ones.

XS142 is a *B. velezensis* strain as the OrthoANI value with the model stain *B. velezensis* FZB42 is 98%. *B. velezensis* FZB42 is used as a biofertilizer and biocontrol agent in agriculture and it is commercially available. This model strain is isolated from sugar beet and colonizes the rhizosphere but not the endophytic compartment (Krebs et al., 1998; Fan et al., 2011). To study the mode of root colonization, XS142 was transformed with a GFP construct (XS142-GFP). This transformant maintained the antagonistic activity like wild type XS142 and showed that XS142 can colonize the interior of potato roots from the root surface to root cortex (Figure 4).

As endophytic bacteria can colonize a similar ecological niche as *V. dahliae*, it has been proposed that they have a better potential to antagonize this pathogen (Eljounaidi et al., 2016). This idea is supported by the observation that an endophytic *Pseudomonas fluorescens* PICF7 strain is an efficient biocontrol agent against *V. dahliae* in olive (Prieto et al., 2009). As XS142 has been isolated from a potato field and is an endophyte, it is very probable that XS142 is a better biocontrol agent for potato Verticillium wilt than FZB42. In our study, we pretreated potato with XS142, before they were inoculated with *V. dahliae*. This method indicates that in future applications in agriculture young potato plants should be inoculated with XS142 before they become infected by *V. dahliae*.

Endophytes are hosted inside the root. Therefore endophyte bacteria are more intimate interaction than the interaction with rhizosphere bacteria and for this reason considered to be more stringently controlled by the host (Rosenblueth and Martínez-Romero, 2006; Weyens et al., 2009). This study makes XS142 an attractive biocontrol agent for potato Verticillium wilt, as it is isolated from a potato field.

It is a great advantage that XS142 is closely related to FZB42, and 8 BGCs of XS142 are shared with this strain (Table 3). Three of them are involved in the production of surfactin, bacillomycin D, and fengycin, respectively. The structure of these compounds made by FZB42 have been analyzed and it has been shown that they have antifungal properties. The BGCs involved in the biosynthesis of bacillomycin D, and fengycin are composed of the same genes and they have a high sequence identity. Therefore it is very probable that the XS142 BCGs, upon expression, will result in the production of compounds with the same structure. However, it remains to be demonstrated that these metabolites are produced when XS142 colonizes potato roots. In case of the surfactin BGC, the one of XS142 lacks 4 genes in comparison to the cluster of FZB42. This BGC includes a regulatory gene involved in controlling the level of surfactin production. Therefore it is less certain to what extend the surfactin BGC can contribute to the antifungal activity of XS142.

Furthermore, FZB42 makes several volatiles with antimicrobial activity. The genome of XS142 harbors the genes for the biosynthesis of 2,3-butanediol and its precursor. It has been shown that 2,3-butanediol can reduce the growth of the bacterial pathogen *Ralstonia solanacearum* (Tahir et al., 2017). However, in a comparative study on the antifungal activity of 9 volatiles made by *Bacillus amyloliquefaciens* L3, 2,3-butanediol was shown to have the lowest antagonistic activity (Wu et al., 2019). So in case XS142 produces 2,3-butanediol, this compound will most likely not contribute a lot to the suppression of *V. dahliae* growth. However, it has been shown, by several studies, to have plant growth promoting activity (Ryu et al., 2003; Shao et al., 2014; Yi et al., 2016; Borriss, 2011). It is noteworthy that XS142 does stimulate potato growth (Figure 5A). So it is possible that production of 2, 3-butanediol contributes to potato growth.

A chitinase gene was found both in XS142 and FZB42. Chitinase is often involved in defense against phytopathogenic fungi by degrading their cell wall (Ni et al., 2018; Kim et al., 2017).

It is likely XS142 has a great potential to directly antagonize *V. dahliae* by producing compounds/enzymes with antifungal properties (Kröber et al., 2014; Dunlap et al., 2019; Gu et al., 2017; Chowdhury et al., 2015; Kaku et al., 2006; Wan et al., 2008; Shao et al., 2014). It remains to be demonstrated which compounds are made when XS142 colonizes potato roots. Under free living conditions XS142 has antagonistic activity against all 4 potato pathogens that we tested, showing that this strain does make antimicrobial compounds. In these plate studies it had, of the 5 strains that we tested the lowest antagonistic against *V. dahliae*. However, in potato XS142 had the highest biocontrol activity. These results strongly suggest that (some) genes involved in the production of antifungal compounds are upregulated when XS142 is hosted in potato roots.

It has been shown that compounds made by FZB42 play a role in ISR (Chowdhury et al., 2015; Fan et al., 2018; Borriss et al., 2019). In these studies, this is the induction of resistance in parts of the plant that have not been in direct contact with the pathogen (previously named SAR). This pathogen induced systemic resistance is not relevant for our study as we want to understand how XS142 suppresses the growth of *V. dahliae* in the root. Some beneficial bacteria can induce another form of ISR (Fan et al., 2018; Wu et al., 2018; Choudhary and Johri, 2009; Pieterse et al., 2014). In this case the beneficial bacteria prime the plant for defense, in the absence of the pathogen. This priming can for example be visualized by exposure of plants to a pathogen which results in an enhanced transcriptome response. Although the terms SAR and ISR are synonymous, we used ISR for priming the plant for defense induced by beneficial bacteria.

We studied whether inoculation with XS142 resulted in ISR in priming. To test this, we used 72 genes that are upregulated as response to *V. dahliae*. In our case, a pretreatment of potato with XS142 did not result in an enhanced transcriptional response upon inoculation with *V. dahliae*. Therefore, we concluded that XS142 cannot induce ISR. To our knowledge only SAR and ISR has been studied for FZB42.

The 72 genes that are induced by *V. dahliae* are most likely part of the defense response of the plant as these genes include well studied defense related genes like WRKY family transcription factor and Defensin (Supplementary Table S1). However, we especially used this gene set to study whether inoculation with XS142 resulted.

In addition to the potential to make antimicrobial compounds XS142 also activates potato genes encoding peptides with antimicrobial activity. This finding is similar to the results of Léon-Kloosterziel et al. (2005). They found that beneficial strains of *Pseudomonas fluorescens* induce in Arabidopsis of *AtTLP1*, which encodes a thaumatin-like protein. Although these bacteria induce ISR, a knockout mutation in this gene has no effect on their ability to induce ISR (Léon-Kloosterziel et al., 2005). Therefore it is likely that induction of *AtTLP1* was a local response of Arabidopsis roots to colonization by *Pseudomonas fluorescens* similar to the induction of potato osmotin genes by XS142.

So XS142 most likely provides some resistance to *V. dahliae* by compounds that suppress its growth and by activation of potato genes encoding peptides with antifungal activity. The transcriptional response of potato to invasion by *V. dahliae* is not enhanced by XS142, it is even decreased. We assume that this is due to the decreased level of *V. dahliae*. So XS142 might become a useful biocontrol agent for potato not only for *V. dahliae* but also for other (important) potato pathogens. It is now important to study the activity of XS142 in the field. As XS142 is isolated from a potato field, it might already have some activity in the field. However, when this activity is too low, XS142 can be a good start to build a SynCom with a higher activity in the field. To build an effective SynCom in future studies, compatibility analysis on roots will be important also in relation to the endogenous soil microbiome.

## Data Availability

The datasets presented in this study can be found in online repositories. The names of the repository/repositories and accession number(s) can be found in the article/Supplementary material.

## Footnotes

^1^ https://github.com/aquaskyline/SOAPdenovo2

^2^ http://ccb.jhu.edu/software/glimmer/index.shtml

^3^ http://eggnogdb.embl.de/#/app/home

^4^ http://www.cazy.org/

^5^ http://www.ezbiocloud.net/sw/oat

^6^ http://bioconductor.org/packages/stats/bioc/DEGSeq/
