# Supplementary material for: Endophytic Bacillus velezensis XS142 is an efficient antagonist for Verticillium wilt of potato
Source: Front Microbiol. 2024 Aug 27;15:1396044. doi: 10.3389/fmicb.2024.1396044 (PMC11385860; doi:10.3389/fmicb.2024.1396044)
Supplement: Supplementary file 1 [file Data_Sheet_1.PDF]

Supplementary Material

Supplementary Figures

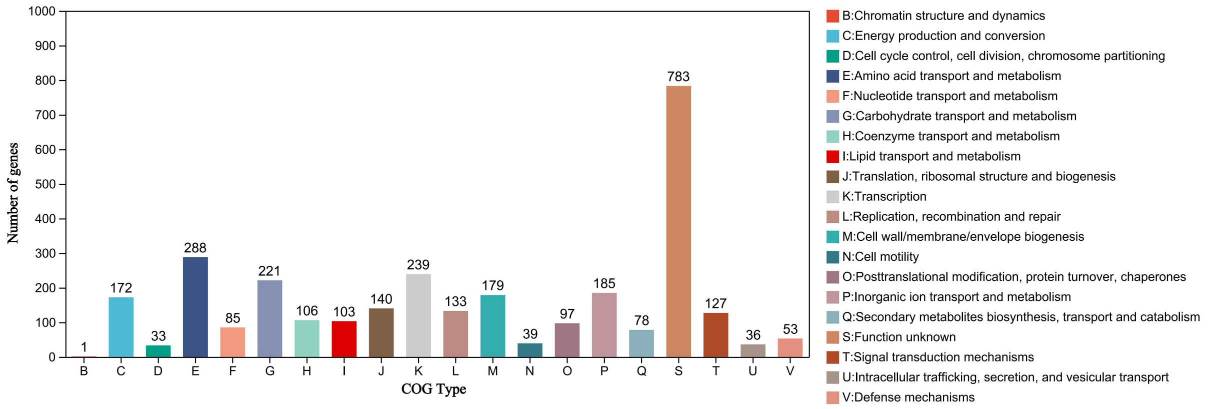

Supplementary Figure 1. COG function classification of XS142. The horizontal coordinate represents different COG types, and the vertical coordinate represents the number of genes. For specific functional descriptions of each COG type, see the legend on the right.

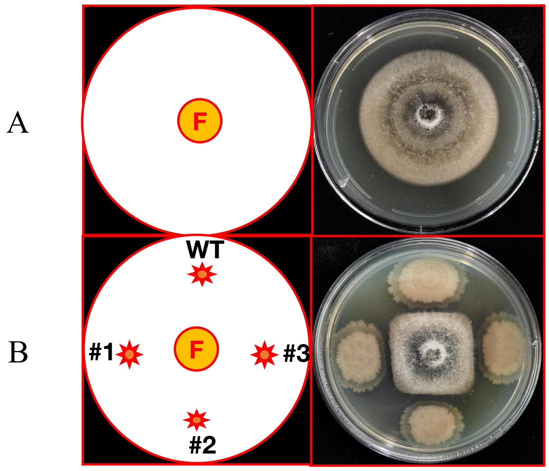

Supplementary Figure 2. Antagonistic activity of positive transformants. (A) Colony morphology of *V. dahliae*. **(B)** Antagonistic activity of wild-type XS142 and positive transformants on *V. dahliae*. F, a fresh mycelial plug (9-mm diameter) of *V. dahliae.* WT, XS142. #1, #2, #3 represent positive transformants of XS142.
